# HPV Prevalence in the Dutch cervical cancer screening population (DuSC study): HPV testing using automated HC2, cobas and Aptima workflows

**DOI:** 10.1186/s12885-016-2961-2

**Published:** 2016-11-28

**Authors:** Cornelis Johannes Jacobus Huijsmans, Willemina Rosalia Rita Geurts-Giele, Cindy Leeijen, Hendrikus Lambertus Cornelius Maria Hazenberg, Jenneke van Beek, Carola de Wild, Johannes Cornelis van der Linden, Adrianus Johannes Christiaan van den Brule

**Affiliations:** 1 Pathologie-DNA, Location Jeroen Bosch Hospital, Henri-Dunantstraat 1, 5223 GZ Den-Bosch, The Netherlands; 2 Department of Pathology, Erasmus MC Cancer Institute, Wytemaweg 80, 3015 CN Rotterdam, The Netherlands; 3 Pathologie-DNA, Location Rijnstate Hospital, Wagnerlaan 55, 6815 AD Arnhem, The Netherlands

**Keywords:** Cervical cancer, Population screening, High risk human papillomavirus, hrHPV prevalence

## Abstract

**Background:**

Primary high risk (hr)HPV screening will be introduced in The Netherlands in January 2017. Our aim was to determine the hrHPV prevalence in the Dutch cervical cancer screening population (DuSC study).

**Methods:**

A total of 12,113 residual PreservCyt cervical samples from the Dutch population based cytology screening program were rendered anonymous, randomized and tested for hrHPV using 3 HPV assays on their respective automated platforms: QIAGEN’s *digene*® HC2 HPV DNA Test® (HC2, signal amplification), Roche Cobas® HPV test (DNA amplification) and Hologic Aptima® HPV Test (RNA amplification). To determine the agreement between results generated using the different assays, pair wise comparison of the systems was performed by determining kappa coefficients.

**Results:**

The selected samples were representative for the population based screening program with respect to age distribution and cytology classification.

HrHPV prevalences found were: 8.5% for HC2 (n = 959), 8.1% for cobas (n = 919) and 7.5% for Aptima (n = 849), resulting in a mean hrHPV prevalence of 8.0 ± 0.5%. Although the hrHPV prevalences of the different assays are in the range of 8%, there was a significant difference in prevalence for the HC2 vs. Aptima assay (p-value = 0.007).

A clear age dependency was found, with an hrHPV prevalence ranging from 18.7 ± 1.2% in women 29-33 years of age to 4.2 ± 0.2% in women 59–63 years of age. Furthermore, a correlation between hrHPV prevalence and severity of cytology was observed, ranging from 5.5 ± 0.4% in normal cytology to 95.2 ± 1.7% in severe dysplasia.

Indeed, kappa coefficients of 0.77, 0.71 and 0.72 (HC2 vs cobas, cobas vs Aptima and Aptima vs HC2, respectively) indicated substantial agreement between the results generated by the different systems. However, looking at the hrHPV positive samples, only 48% of the samples tested positive with all 3 assays.

**Conclusions:**

A hrHPV prevalence of 8% was found in this unselected population based screening cohort independently of using HC2, Aptima or cobas. This prevalence is higher than the previously reported 4–5% (POBASCAM and VUSA-Screen trials). Furthermore, the complete automated hrHPV detection workflow solutions from QIAGEN, Roche, and Hologic were successfully used and will be valuable for reliably implementing high throughput hrHPV testing in cervical cancer screening.

## Background

Since the 1980s, a well monitored cervical cancer screening program has been employed in The Netherlands, which was further improved by implementing high risk human papillomavirus (hrHPV) triage in 2006. Women aged 30–60 years are invited to undergo screening once every five years. The screening consists of a Pap smear (liquid based cytology) which is examined by microscopy for the presence of abnormal cells. Depending on the outcome: 1) referral takes place to the next screening round (no abnormalities), 2) molecular triage testing takes place for the presence of hrHPV (equivocal or mild abnormalities) and 3) immediate referral to the gynaecologist takes place (moderate or severe abnormalities). When hrHPV is present in cases of equivocal or mild abnormalities, gynaecologist referral takes place; when hrHPV is absent the patient is invited for follow up cytology testing in 6 months.

Although the current screening program has been shown to be effective in reducing the incidence of cervical cancer and the number of cervical cancer related deaths, cervical cancer is still substantially missed and there is potential for improvement [1]. Therefore, based on a recommendation by the Dutch health council, primary cytology screening will be replaced by primary hrHPV screening.

Primary hrHPV screening with molecular testing has been shown to be more sensitive in detecting high-grade cervical intraepithelial neoplasia (CIN2-3) [2]. This higher sensitivity makes it possible to reduce the number of screening rounds. In case of the Dutch situation, the number of screening rounds will decrease from 7 to 5 (30, 35, 40, 50 and 60 years) [1–3]. However, due to lower clinical specificity, hrHPV testing might lead to the detection of clinically irrelevant hrHPV infections leading to increased referral to the gynaecologist and unnecessary colposcopy [4]. Nowadays many hrHPV tests are commercially available. These assays differ significantly with regard to analytical and clinical sensitivities and specificities and are therefore not all suitable for use in population based screening. Therefore, in 2009 international guidelines were established for human papillomavirus DNA test requirements for primary cervical cancer screening in women 30 years and older [5, 6].

Modeling has indicated that the new Dutch cervical cancer screening program design, based on primary hrHPV testing, will prevent around 11% (n = 75 out of 700) more of annual Dutch cervical cancer cases and 7–9% (n = 18 out of 200–250) more of annual cervical cancer related deaths in the Netherlands, whilst maintaining the costs of the current screening program [1]. With regard to the cost-effectiveness of such a program, hrHPV prevalence within the screening cohort, amongst other costs of the hrHPV test, is of pivotal importance. Based on the POBASCAM and VUSA screen studies, the Dutch health council stated an overall hrHPV prevalence of 4–5% within the population of the cervical cancer screening program [2, 7].

Several large studies have been performed to date regarding the efficacy of primary hrHPV screening, hrHPV prevalence and comparison of hrHPV detection technologies [8–12]. The number of samples in these studies ranged from 6,000 to over 44,000 and were derived from (a selection of) women from different cervical cancer screening cohorts. The employed hrHPV detection assays were GP5+/6 + PCR-EIA, Hybrid Capture 2, Aptima and cobas which yielded hrHPV prevalences ranging from 3.6% to 16.2%.

None of these studies, however, were performed in a setting exactly simulating the current (Dutch) population based screening program but often in a well defined study population. Additionally, no studies have yet been conducted using the currently available, fully automated – including de- and recapping and processing of primary sample containers - hrHPV testing systems capable of processing the large sample numbers involved in a primary screening setting. Our aim was therefore to determine hrHPV prevalence in the DuSC (**Du**tch **s**creening **c**omparison) hrHPV study, investigating a cohort representative for the Dutch population based screening cohort using fully automated hrHPV solutions that are currently commercially available and use hrHPV detection assays that were approved by the Dutch Society of Pathology and International guidelines for HPV testing [5, 6, 13].

## Methods

### Study population and design

Residual ThinPrep® PreservCyt® Collection Medium (Hologic, Marlborough, MA) with cervical samples from the Dutch population based cytology screening program from the Eastern part of the Netherlands were used. In total, 12,113 consecutive samples from August 2013 to July 2014 were included from women with a “primary invitation” for the following screening round, thereby excluding follow-up samples. Age distribution was matched with that of the Dutch screening population based on the number of Dutch women in each age group in combination with the attendance rates per age group [14]. Age groups were based on the invitation schedule for the screening program and defined as follows: 29–33, 34–38, 39–43, 44–48, 49–53, 54–58 and 59–63 years of age. Because all included samples were derived from the primary screening population, matched age distribution based on the actual intake of the Dutch screening program, was expected to also result in a representative distribution of cytology classification. Following inclusion, the samples were randomized, and rendered anonymous using barcodes randomly generated by the employed Access database. The bar-coded containers were suitable for further automated sample handling. Dutch regulation does not allow use of residual samples from the screening program aged <4 months. Therefore, minimal sample age at time of testing was 4 months and ranged up to 12 months. Consequently, all included samples exceeded the manufacturers’ storage recommendation of 3 months. Further storage conditions were as recommended by the manufacturer.

All included samples were tested for hrHPV using 3 fully automated testing solutions – from primary sample processing to interpretation of results - that are currently available and use hrHPV tests approved by the Dutch Society of Pathology (NVVP [15]) and international guidelines for hrHPV testing [5, 6, 13]. These were: the *digene* HC2 High-Risk HPV DNA Test® (HC2; Qiagen, Gaithersburg, MD), the cobas® HPV Test (cobas; Roche Diagnostics, Pleasanton, CA) and the Aptima® HPV Test (Aptima; Hologic, San Diego, CA). Specifications of each test are depicted in Table 1.

**Table 1:** Overview of the HC2, cobas and Aptima test specifications and accompanying automated testing solutions

Assay	Nr. of devices	HrHPV types detected	Target gene	Detection technology	Input volume	Dead volume	Control
HC2	4	16/18/31/33/35/39/45/51/52/56/58/59/68	Entire hrHPV genome (DNA)	Nucleic acid hybridization assay with signal amplification	3.0 mL	5.0 mL	Process control for each sample batch
cobas	3	16/18/31/33/35/39/45/51/52/56/58/59/66/68	*L1* (DNA)	Real time Polymerase Chain Reaction	0.25 mL	0.5 mL	ß-globin for process validity of individual sample and input sufficiency
Aptima	2	16/18/31/33/35/39/45/51/52/56/58/59/66/68	*E6/E7* (RNA)	Transcription Mediated Amplification	0.4 mL	5.0 mL	Spiked internal control for process validity of individual sample

*HC2* Hybrid Capture 2

Dedicated laboratory staff was trained by all 3 manufacturers and certified to perform the standardized protocol for each system. A rotation scheme was employed resulting in different testing orders (HC2- cobas - Aptima, cobas-Aptima-HC2, Aptima-HC2-cobas etc.) to prevent an influence on test performance caused by the order in which the samples were analyzed by the different hrHPV workflows.

### digene® HC2 High Risk HPV DNA Test® (HC2)

The HC2 solution uses the *digene®* HC2 High Risk HPV DNA Test® (QIAGEN, Gaithersburg, MD), which is based on signal amplification using RNA probes to target the entire hrHPV genome. Samples were placed in the M1 decapper system where barcode identification, homogenization, decapping/recapping and sample transfer to a bar-coded tube took place. Subsequently, automated sample preparation of cervical cells was performed using the QIAsymphony DSP HPV Media Kit in combination with the QIAsymphony. Resulting sample extracts were tested for hrHPV using the *digene®* HC2 High-Risk HPV DNA Test*®* with the Rapid Capture System. Automated amplified chemiluminescent signal detection and results reporting (hrHPV positive: relative light unit per cut off value (RLU/CO) ≥1.0; hrHPV negative RLU/CO < 1.0) was performed using the DML3000 luminometer. All steps were performed according to the manufacturer’s protocols.

### cobas® HPV Test (cobas)

The cobas® HPV Test from Roche (Roche Diagnostics, Pleasanton, CA) is a real time PCR based assay targeting the hrHPV L1 gene and the human ß-globin gene as an internal control. Uracil N-glycosylase (UNG) and dUTP are utilized to eliminate possible amplicon carryover. After barcode identification, homogenization and decapping of the PreservCyt vials using the p480 instrument, the samples were transferred to the x480 system for DNA extraction and real time PCR setup using the Sample Preparation Kit (c4800 SMPL PREP) and Liquid Cytology Preparation Kit (c4800 LIQ CYT). Samples were homogenized again by automatic pipetting prior to extraction. Processed sample vials were transferred back to the p480 system for recapping. Amplification, detection and hrHPV typing (types 16, 18 and 12 “other” hrHPV types) by real time PCR and results reporting (hrHPV positive: Cp value <40.0 for hrHPV 18 and 12 other genotypes and Cp-value <40.5 for hrHPV 16; hrHPV negative Cp value >40 and >40.5, respectively) was performed using the z480 in combination with the HPV Amplification/Detection Kit (c4800 HPV AMP/DET). All steps were performed according to the manufacturer’s protocols.

### Aptima® HPV Test (Aptima)

The Aptima® HPV Test (Hologic/Gen-Probe, San Diego, CA) relies on mRNA amplification targeting the E6/E7 genes. Sample identification, homogenization, decapping/recapping and transfer to a bar-coded Aptima tube with pierceable cap was done using the Tomcat system. The Aptima tube was transferred to the Panther system for specific capture of hrHPV E6/E7 mRNA transcripts by oligomer coated magnetic particles, transcription mediated amplification, detection and results reporting (hrHPV positive: RLU/CO ≥ 0.5; hrHPV negative RLU/CO < 0.5) using the Aptima HPV assay. The assay incorporates a spiked internal control monitoring mRNA capture, amplification and detection, as well as operator and instrument error. When sufficient remnant sample was available (volume used 0.4 mL, dead volume 5 mL), genotyping (types 16 and 18/45) of hrHPV positives was subsequently performed using the Aptima® HPV 16 18/45 Genotype Assay. All steps were performed according to the manufacturer’s protocols.

### Checkerboard

To exclude a significant impact of sample contamination on the hrHPV prevalence found in our study, we performed a checkerboard experiment with all three systems.

Ninety-four residual PreservCyt samples from our routine hrHPV diagnostics (hrHPV triage testing) with a high hrHPV viral load were pooled to a volume of 1.4 L. This pool was mixed thoroughly and aliquoted in 3 sets of 44 empty PreservCyt containers in portions of 8 mL per container. Each set was designated to 1 hrHPV testing system and alternately tested with 44 containers with clean PreservCyt solution. Testing of these checkerboard samples was performed as with diagnostic samples, starting from the processing of the cervical scrape to (signal)amplification and interpretation of results.

To ensure that this pool contained a sufficient amount of hrHPV, the Cp value was determined with cobas and compared to the Cp values of all hrHPV positives included in the study.

### Statistical analysis

The overall agreement for each combination of 2 hrHPV tests was determined by calculation of Cohen’s Unweighted Kappa coefficient (ĸ-coefficient). An agreement was considered to be substantial with a ĸ-coefficient of ≥0.6 [16]. Inter-assay and pair wise agreements were calculated as the total number of samples in a group that were hrHPV positive on at least one of the systems compared, divided by the number of samples that were hrHPV positive on all compared systems.

## Results

### Study population

A total of 12,113 residual PreservCyt cervical samples were randomized, rendered anonymous and included from the Dutch population based cytology screening program (district East).

**Fig. 1:**
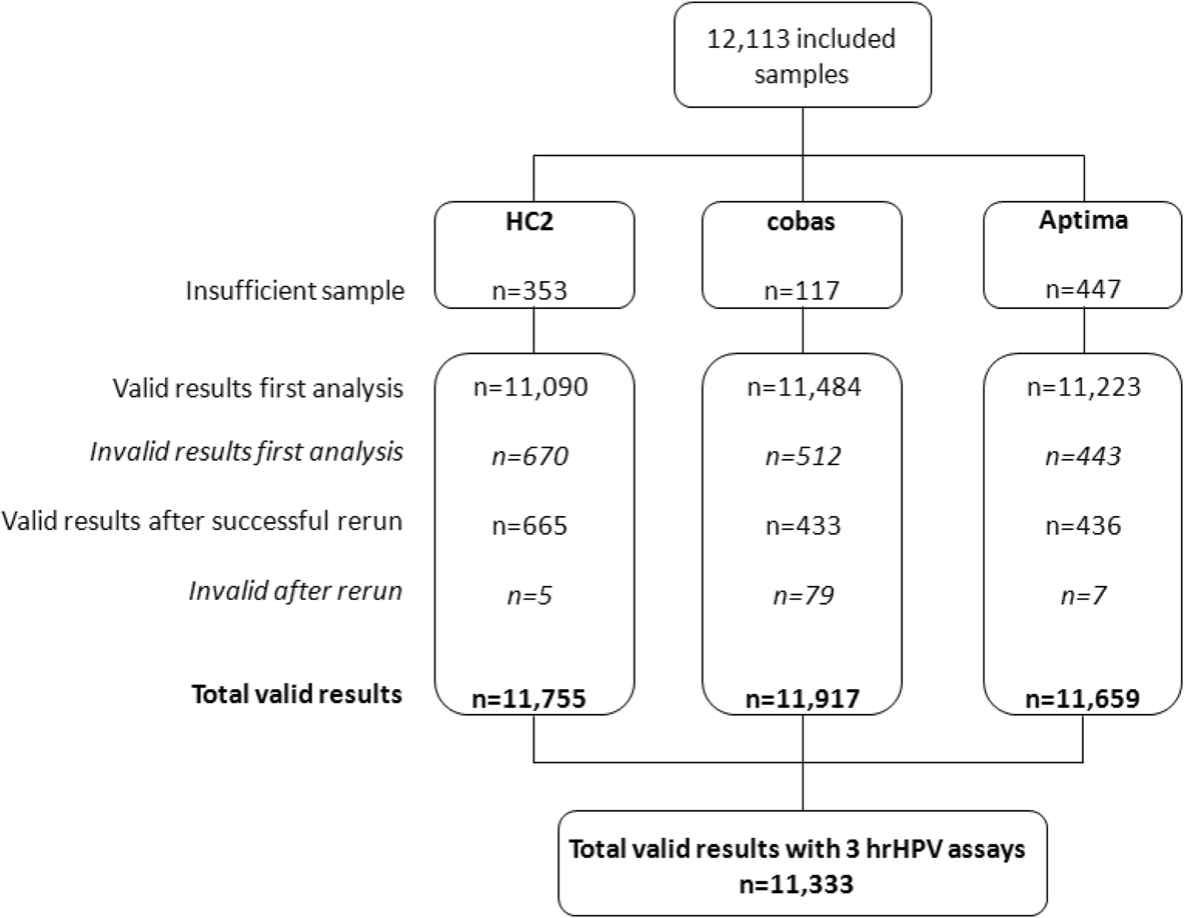
Flowchart of included sample numbers. Number of valid results vs invalid results per assay and with all 3 assays. Invalid may be due a technical run error, inhibition, sampling error etc

Sample volumes used per system were 0.25 mL, 3.0 mL and 0.4 mL for cobas, HC2 and Aptima, respectively. Dead volumes per system were 0.5 mL for cobas, 5.0 mL for HC2 and 5.0 mL for Aptima. The total sample volume required therefore amounted to 8.65 mL (3.65 mL used and 5.0 mL dead volume). As a result, sufficient volume was available for 11,755, 11,917 and 11,659 samples to be tested with HC2, cobas and Aptima, respectively. Results were considered invalid in cases that the reporting software flagged a sample result with “error” or “invalid”. Underlying reasons for such flags could not be further investigated due the way the systems generated the respective output files but are for example technical run errors (affecting an entire run or a large part of a run), inhibition, sampling errors due to clotting of pipet tips etc. For each assay, a number of these invalid results were found of which the correspondent samples were retested: HC2 n = 670, cobas n = 512 and Aptima n = 443, which was successful in n = 665, n = 433 and n = 436 samples, respectively. In n = 5, n = 79 and n = 7 samples, results remained invalid. The substantial difference in initial invalids versus repeat invalids can be largely explained by the fact that in the case of a technical run error, simply repeating the run resulted in valid results for the respective sample set. In total, from 11,333 out of 12,113 samples, hrHPV analysis generated valid results on all 3 systems (Fig. 1). All further analyses were performed on the sample group that could be tested by all three assays. Distribution of age and cytology classification of this group were checked and found to be identical the Dutch screening population (see Tables 2 and 3).

**Table 2:** Age distribution of included patients of which test results of all 3 assays are available (n = 11,333).

Age (years)	Included samples (N)	Age distribution of tested cohort (DuSC)	Age distribution of Dutch screening population	HC2 hrHPV prevalence %(N)	cobas hrHPV prevalence %(N)	Aptima hrHPV prevalence %(N)	Overall hrHPV prevalence
29–33	1,191	10.5%	10.5%	19.1% (228)	19.7% (235)	17.3% (206)	18.7 ± 1.2%
34–38	1,317	11.6%	11.7%	11.7% (154)	11.5% (152)	10.6% (140)	11.3 ± 0.6%
39–43	1,775	15.7%	16.3%	9.0% (159)	8.0% (142)	7.3% (130)	8.1 ± 0.9%
44–48	1,928	17.0%	16.9%	7.7% (148)	7.1% (137)	6.3% (122)	7.0 ± 0.7%
49–53	1,870	16.5%	16.4%	6.3% (117)	5.4% (101)	5.4% (101)	5.7 ± 0.5%
54–58	1,703	15.0%	14.7%	5.4% (92)	5.0% (86)	4.9% (84)	5.1 ± 0.3%
59–63	1,549	13.7%	13.6%	3.9% (61)	4.3% (66)	4.3% (66)	4.2 ± 0.2%
Total (N)	11,333			959	919	849	

Number of included samples per age group and age distribution of study cohort vs age distribution of the Dutch screening program; and hrHPV prevalences per assay and overall mean

**Table 3:** Pap class distribution of included patients of which test results of all 3 assays are available (n = 11,333).

Pap class	Bethesda classification	Included samples (N)	Pap class distribution of tested cohort (DuSC)	Pap class distribution of Dutch screening population^a^	HC2 hrHPV prevalence %(N)	cobas hrHPV prevalence %(N)	Aptima hrHPV prevalence %(N)	Overall hrHPV prevalence
0	-	191	1.7%	1.7%	6.8% (13)	5.2% (10)	4.2% (8)	5.4 ± 1.3%
1	Normal	10,639	93.9%	93.6%	5.8% (620)	5.7% (609)	5.1% (545)	5.5 ± 0.4%
2	ASC-US	314	2.8%	3.0%	47.8% (150)	41.1% (129)	39.5% (124)	42.8 ± 4.4%
3a1	LSIL	71	0.6%	0.7%	91.5% (65)	85.9% (61)	84.5% (60)	87.3 ± 3.7%
3a2	HSIL (moderate)	50	0.4%	0.4%	94.0% (47)	94.0% (47)	94.0% (47)	94.0 ± 0.0%
3b	HSIL (severe)	62	0.5%	0.6%	95.2% (59)	93.5% (58)	96.8% (60)	95.2% ± 1.7%
4	CIS/AIS	6	0.01%	0.0%	83.3% (5)	83.3% (5)	83.3% (5)	83.3 ± 0.0%
	Total (N)	11,333			959	919	849	

Number of included samples per Pap class and Pap class distribution of study cohort vs Pap class distribution of the Dutch screening program; and hrHPV prevalences per assay and overall mean

Pap 0 = sample inadequate for cytology

^a^Based on the Dutch national pathology registry (PALGA database)

*ASC-US* Atypical Squamous Cells of Undetermined Significance, *LSIL* Low-grade Squamous Intraepithelial Lesion, *HSIL* High-grade Squamous Intraepithelial Lesion, *CIS* Carcinoma in situ, *AIS* Adenocarcinoma in situ

### HrHPV prevalence

Performing the analysis based on the group of samples tested by all 3 systems (n = 11,333), hrHPV prevalences found were 8.5%, 8.1% and 7.5% for HC2, cobas and Aptima, respectively. Overall prevalence was found to be 8.0 ± 0.5%. Because of this rather low deviation, the overall or mean hrHPV prevalence of the 3 assays is considered to more closely resemble the “true” prevalence. Therefore, the overall hrHPV prevalence is presented throughout the manuscript, unless indicated otherwise.

If the cumulative hrHPV prevalences for the entire cohort, i.e. all age groups and all cytology classes, were determined based on the maximum amount of samples analyzed with each assay (HC2 n = 11,755; cobas n = 11,917; Aptima n = 11,659) these were found to be 8.5%, 8.1% and 7.6%, respectively. Resulting in no statistical significant difference when compared to the group of samples tested by all 3 systems.

The rotation scheme used to prevent an influence on assay performance by the order in which the samples were analyzed by the different hrHPV workflows, resulted in hrHPV prevalences of 8.0–8.9%, 7.6–8.6% and 7.0–7.9% for HC2, cobas and Aptima, respectively, and were not significantly different within a particular assay (*p*-values range from 0.25–0.28) and differences observed are due to different age distribution of the subgroups.

**Fig. 2:**
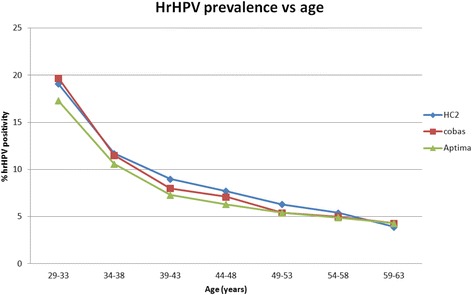
HrHPV prevalence vs. age for HC2, cobas and Aptima. Mean hrHPV prevalence per age group conform the Dutch cervical cancer screening program for all cytology classifications. HC2 = Hybrid Capture 2

**Fig. 3:**
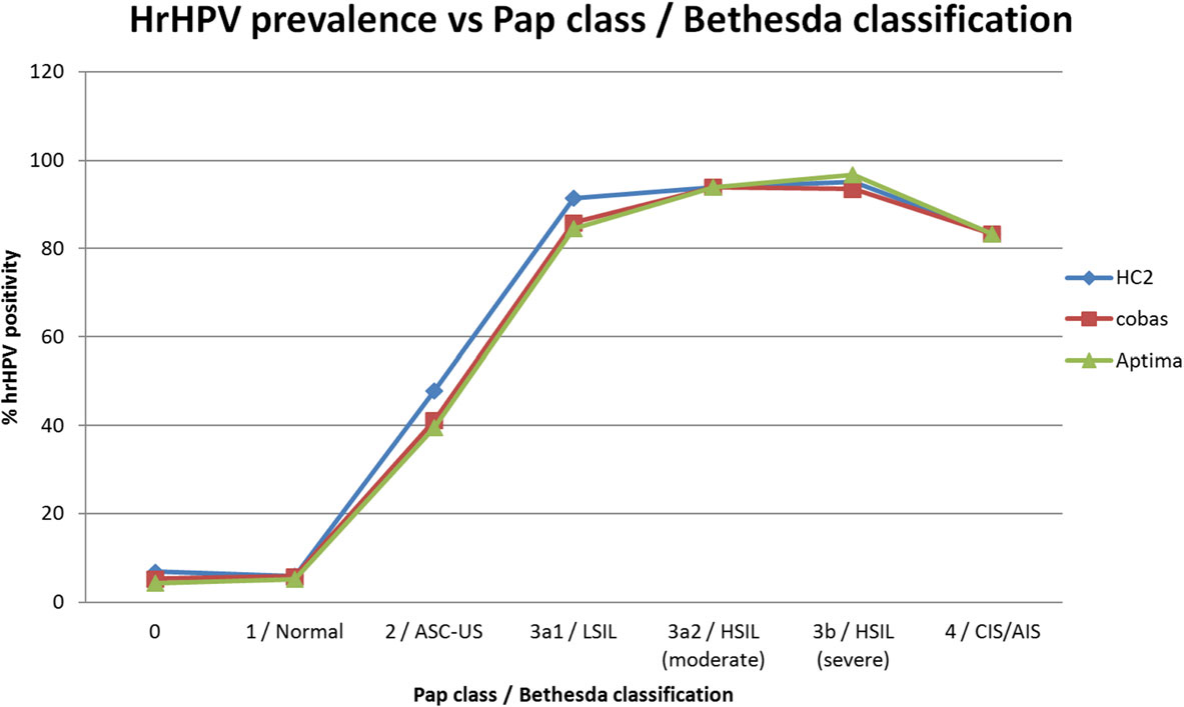
HrHPV prevalence vs. Pap class for HC2, cobas and Aptima. Mean hrHPV prevalence per Pap class for all ages. HC2 = Hybrid Capture 2. ASC-US = Atypical Squamous Cells of Undetermined Significance; LSIL = Low-grade Squamous Intraepithelial Lesion; HSIL = High-grade Squamous Intraepithelial Lesion; CIS = Carcinoma in situ; AIS = Adenocarcinoma in situ

For each hrHPV assay, the hrHPV prevalence was also determined per age group (Fig. 2, Table 2) and Pap class (Fig. 3, Table 3).

The hrHPV prevalence decreased with an increasing age of the women involved, from 18.7 ± 1.2% in women aged 29–33 years to 4.2 ± 0.2% in women 59–63 years of age. As for severity of cytology, a correlation was also observed with hrHPV prevalence, ranging from 5.5 ± 0.4% in normal cytology to 95.2 ± 1.7% in severe dysplasia.

### Checkerboard

The hrHPV pool used for the checkerboard generated mean Cp values using cobas of 23.83 ± 0.5, 25.96 ± 0.79 and 23.14 ± 0.86 for hrHPV16, hrHPV18 and “other” hrHPV types, respectively. When compared to the Cp values of all hrHPV positive samples included in our study, the hrHPV concentration in the pool is found to be representative for 12.6%, 15.7% and 12.6% of the most highly positives study samples containing hrHPV16, hrHPV18 and “other” hrHPV types, respectively. All 44 samples containing clean PreservCyt solution generated a hrHPV negative result with the HC2, cobas and Aptima system, therefore no contamination was observed.

### HrHPV assay comparison

Kappa-coefficients of each combination of 2 hrHPV tests were based on the number of samples that generated results using the 3 systems (n = 11,333) and were as follows: HC2 vs cobas 0.77; cobas vs Aptima 0.71; and Aptima vs HC2 0.72.

**Fig. 4:**
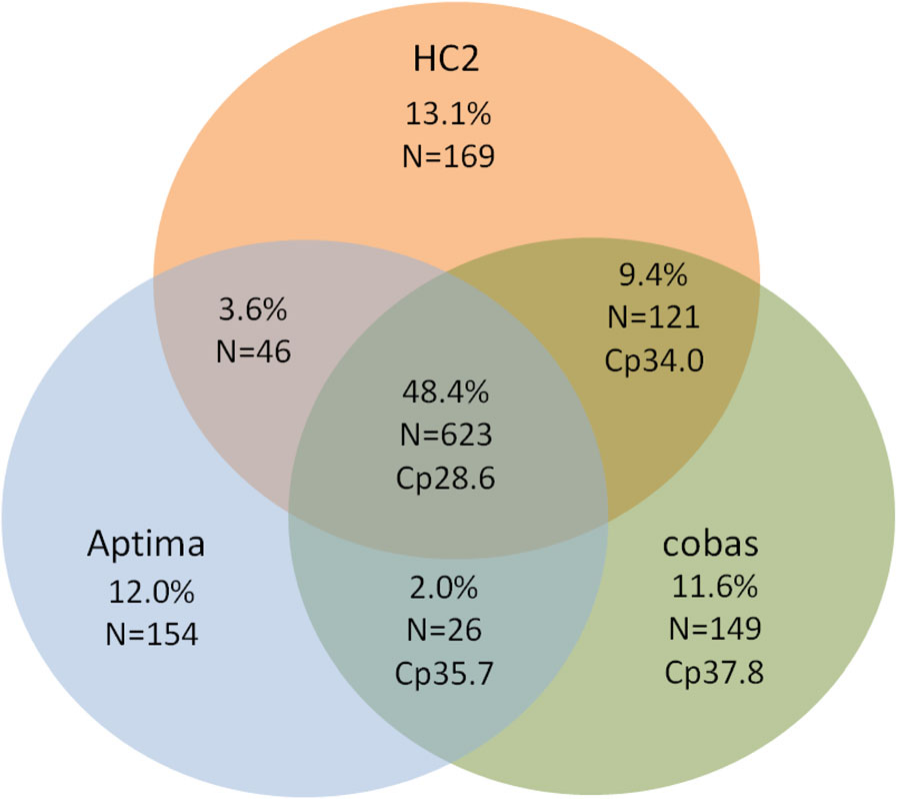
Inter-assay agreement between HC2, cobas and Aptima. Each proportion was calculated based on the total number samples found hrHPV positive (n = 1,288) in at least one of the assays

A total of 1,288 samples were found to be hrHPV positive with at least one system, of which 48.4% (n = 623) was hrHPV positive in 3 out of 3 systems, 15.0% (n = 193) in 2 out of 3 systems and 36.6% (n = 472) in 1 out of 3 systems (Fig. 4). Mean cobas Cp values generated in these groups were Cp 28.6 (HC2, Aptima and cobas), 34.0 (HC2 and cobas) and 35.7 (Aptima and cobas) and Cp 37.9 (single positives only found by cobas), respectively (Fig. 4). Inter-assay agreement ranged from 65.7% in women aged 29–33 years to 31.8% in women 59–63 years of age (Table 4). For severity of cytology the inter-assay agreement ranged from 36.2% in Pap 1 to 91.8% and 100% in Pap 3b and 4, respectively (Table 4). Of the hrHPV positive samples with abnormal cytology (Pap 2/ASC-US and higher), 83.8% was found to be positive in 3 out 3 systems, whereas 7.2% was hrHPV positive in 1 out of 3 systems. On the contrary and as expected, of the hrHPV positive samples with normal cytology (Pap 1), 36.2% and 46.7% were found to be hrHPV positive in 3/3 and 1/3 systems, respectively.

**Table 4:** Inter-assay agreement (number of samples positive in all 3 hrHPV assays; total n = 623) for each age group and cytology classification

Age group	Inter-assay agreement % (N)	Pap class	Bethesda classification	Inter-assay agreement % (N)
29–33	65.7% (180)	0	-	26.3% (5)
34–38	52.0% (105)	1	Normal	36.2% (339)
39–43	51.0% (103)	2	ASC-US	73.4% (113)
44–48	41.7% (86)	3a1	LSIL	90.8% (59)
49–53	47.7% (72)	3a2	HSIL (moderate)	95.8% (46)
54–58	29.5% (43)	3b	HSIL (severe)	91.8% (56)
59–63	31.8% (34)	4	CIS/AIS	100% (5)
Total (N)	623			623

Pap 0 = sample inadequate for cytology

*ASC-US* Atypical Squamous Cells of Undetermined Significance, *LSIL* Low-grade Squamous Intraepithelial Lesion, *HSIL* High-grade Squamous Intraepithelial Lesion, *CIS* Carcinoma in situ, *AIS* Adenocarcinoma in situ

Agreement of hrHPV positives of the Aptima assay, detecting mRNA, when pair wise compared to cobas and HC2, both detecting DNA, was 58.0% (n = 649) and 58.7% (n = 669), respectively. This pair wise agreement was 65.6% (n = 744) for cobas vs. HC2.

In total, 609 of the samples found hrHPV positive by both cobas as well as Aptima could be genotyped by the typing assays of both manufacturers. The outcome of genotyping of hrHPV16, 18, 18/45 and “other” is depicted in Table 5.

**Table 5:** Genotyping outcome from samples positive for hrHPV by cobas and Aptima, N(%)

Assay	hrHPV 16	hrHPV 18	hrHPV 18/45	Other hrHPV types	Total genotyped
cobas	207 (34.0)	64 (10.5)	NA	476 (78.2)	609
Aptima	187 (30.7)	NA	67 (11.0)	370 (60.8)^a^	609

*NA* not applicable

^a^Based on the combination of a positive hrHPV test and the absence of hrHPV16 and 18/45

Prevalence of hrHPV16 was 34.0% (n = 207) in cobas and 30.7% (n = 187) in Aptima, whereas hrHPV18 and hrHPV18/45 were found in 10.5% (n = 64) and 11.0% (n = 67) of typed cases, respectively. In 78.2% (n = 476), cobas typing yielded “other” hrHPV genotypes, whilst Aptima typing suggested that hrHPV genotypes other than 16 and 18/45 were present in 60.8% (n = 370).

## Discussion

### HrHPV prevalence

The presented study, i.e. DuSC study, is the first investigating hrHPV prevalence in a setting representative for the Dutch population based cervical cancer screening program, without any selection of women, and utilized the most recently introduced, fully automated hrHPV testing systems as would be used when primary hrHPV screening is implemented. Cumulative hrHPV prevalences for HC2, cobas and Aptima were in the same range and, based on the sample group yielding results using all 3 assays (n = 11,333), resulted in a mean hrHPV prevalence of 8.0 ± 0.5%. As expected, a correlation was found between hrHPV prevalence and the age of the involved women as well as the Pap class (Figs. 2 and 3), which confirmed the reliability of the blinded hrHPV testing. The hrHPV prevalence in our study population decreased with an increasing age, and was higher when severity of cervical dysplasia increased. The hrHPV prevalence observed in cytological carcinoma in situ (Pap 4) was lower than that of Pap 3a1, 3a2 and 3b, however, the number of samples in the Pap 4 group was too low to be representative (n = 6).

The detected hrHPV prevalence is significantly higher than the 4–5% stated by the Dutch Health Council [1]. The prevalence stated by the Dutch Health council was based on data from the POBASCAM and VUSA screen studies [2, 7]. These studies however, were not designed to determine hrHPV prevalence in the Dutch population based screening setting, but were conducted to evaluate the effectiveness of different triage algorithms (VUSA screen) and primary hrHPV screening (POBASCAM). For example, in both these studies in contrast to our DuSC study, a number of women were excluded from enrollment when they had abnormal cytology or a CIN lesion within the preceding 2 years, most likely resulting in a selection bias. In the setting of the Dutch screening program, women having either equivocal or mild cervical dysplasia (Pap 2/3a1) on such a previous smear would not have been excluded, but would have actually been invited for follow-up after 6 months. This will increase the hrHPV prevalence in the screening cohort by 0.5–1% and partly explains the difference in prevalence when compared to this study. Other explanations for the remaining difference in hrHPV prevalence might be a cohort effect, the fact that POBASCAM and DuSC used different hrHPV assays and/or technical issues as is for example the case in the POBASCAM where crude cell extracts are used instead of purified DNA. Although a different age distribution of the population tested could also be an explanation for the difference in hrHPV prevalence, the women in POBASCAM were in fact younger than in DuSC, which would result in a higher instead of lower hrHPV prevalence in POBASCAM. When comparing our hrHPV prevalence data to other studies published investigating population based screening cohorts, also higher hrHPV prevalence rates were found. Although performed in a population of different, predominantly Canadian, geographical origin and a somewhat different age distribution, hrHPV prevalence found in the population based HPV FOCAL study was similar in women ≥30 years of age with 7.2% and 6.9% for cobas and HC2, respectively [9, 17]. In the ATHENA trial, when excluding patients <30 years, overall hrHPV prevalence found was 8.4% (10.5% including women 25–29 years) [12]. In women from the Danish cervical cancer screening program, the hrHPV prevalence was even higher: 9.4% for Aptima, 11.7% for HC2 and 16.2% for cobas [18]. As earlier described, the hrHPV prevalences found in our study with the 3 different testing solutions were in the same range, although the hrHPV prevalence found by the Aptima assay was found to differ significantly when compared to HC2 (7.5% vs 8.5%, respectively; p-value 0.007). This suggests that with respect to hrHPV prevalence, the performance of all three of the hrHPV tests employed in our study and using fully automated systems, are similar. Additional performance comparison on several technical aspects and the degree of user friendliness per system is not within the scope of this study, but will be described elsewhere (manuscript in preparation).

In theory, the higher than expected hrHPV prevalence found in our study could be influenced by false positive results generated as a result of sample handling; however this is not the case since checkerboard experiments showed no cross contamination between samples. In addition, an influence on test performance caused by the order of hrHPV systems in which the samples were analyzed was ruled out, since samples were randomly assigned to a particular sequence of the 3 hrHPV testing solutions. These different subsets yielded similar hrHPV prevalences, also indicating no significant contamination in a specific testing solution.

Moreover, because of the retrospective nature of this study, sample ages ranged from 4 to 12 months meaning all included samples exceeded the manufacturers’ storage recommendation of 3 months. However, no substantial differences in hrHPV prevalence were observed when comparing different subsets composed of these various sample ages, suggesting no significant effect of sample age on assay performance. This is supported by the fact that repeat analysis of similarly stored samples (n = 153) previously tested for routine hrHPV diagnostics (hrHPV triage) using all three hrHPV detection systems, yielded a concordance of 85% with results previously found and most discrepancies were, as expected, found in weak hrHPV positive samples (data not shown). It should be noted that samples from such a triage setting may harbor a relatively high hrHPV viral load in comparison to screening samples which possibly affected the outcome of stability testing. Therefore an effect on test performance using residual samples, instead of prospective hrHPV testing, cannot be fully excluded. If such an effect would be present, our study design might even lead to underestimation of the hrHPV prevalence due to DNA/RNA degradation in stored residual samples.

So based on hrHPV data presented in this DuSC study, it is realistic to conclude that the hrHPV prevalence in the Dutch population based screening cohort is higher than previously reported, and that hrHPV prevalences from POBASCAM and VUSA-screen are under representing the hrHPV prevalence in the current general population. An higher hrHPV prevalence could lead to an increased referral to the gynecologist and unnecessary colposcopy [3], influencing the health economics of the screening program. Besides the hrHPV prevalence, also other factors such as costs of hrHPV test and screening organization (invitation, logistics, etc) are of major importance. Furthermore, it is very important to have good quality cytology and standardized criteria to circumvent unnecessary referral to colposcopy.

### HrHPV assay comparison

A substantial agreement between the 3 different hrHPV tests used, was found (kappa coefficients ranged from 0.71 to 0.77 when comparing each combination of 2 hrHPV tests). However, despite this good concordance of 3 assays all fulfilling the international guidelines for primary hrHPV screening, a significant number of discrepancies were observed. This finding is in line with several previous studies [19–25]. Because of the large number of hrHPV negatives, strongly influencing ĸ-coefficient and therefore overall agreement, the inter-assay agreement should also be determined based on a comparison of the hrHPV positive samples (Fig. 4). Approximately half (48.4%; n = 623) of the positive hrHPV samples (n = 1,288) generated positive results in all 3 systems. Inter-assay agreement in our study was found to decrease with an increasing age of the women involved (Table 4). Most likely, this is due to the hrHPV viral load which was previously observed to be higher in younger women [26]. This is supported by the fact that Cp values generated by cobas were lowest in the samples found hrHPV positive in 3 out of 3 assays followed by 2 out of 3 assays and single positives (Fig. 4). Also, a correlation was observed between severity of cytology and inter-assay agreement, with an increasing agreement in the more severe abnormalities (Table 4). The DNA assays (HC2 and cobas) show a better concordance than the Aptima mRNA assay. This is also indicated by the lower ĸ-coefficient for the mRNA assay vs. the DNA assays. In theory, this could be due to decreased stability of mRNA in archival samples in comparison to DNA as a target molecule. However, it is more likely the cause of differences in assay specificity that may be higher in mRNA based detection, which can subsequently yield benefits on a population based screening system [27]. In the Horizon study, inter-assay agreement was slightly lower with 37.6% (to be able to compare results, samples only hrHPV positive in CLART were excluded) [24]. Moreover, in the Horizon study, hrHPV detection by cobas generated a relatively high number (30.6%) of hrHPV positives only found by cobas (corrected for samples that were only hrHPV positive in the CLART assay). Although not fully elucidating this observation, there might be an effect of the media used, being SurePath in the Horizon study whereas PreservCyt was used in our study [9, 10, 17, 24]. Interestingly, this relatively large difference between HC2 and cobas was not observed in a recent study, where comparison of these hrHPV tests in women with ASC-US (atypical squamous cells of unknown significance) did not yield significant differences [28]. This observation might be influenced by the fact that the recent study utilized samples from a triage population. Given the higher hrHPV prevalence and possibly higher viral load in a triage population, one may expect an increased inter-assay agreement.

Comparing the hrHPV genotyping results of cobas and Aptima could only be partly done due to the difference in typing assay setup with hrHPV typing of 16, 18 and “other” by cobas and 16 and 18/45 by Aptima. However, hrHPV 16 prevalence was similar with cobas as with Aptima. Moreover, prevalences of hrHPV 16, 18 and “other” found in our study were similar to those found by cobas in the Horizon study [10].

It is of particular interest from a clinical perspective, which of the hrHPV positives found in our study (i.e. certain discrepant samples) are most representative for developing CIN and are therefore of clinical relevance. However, due to the retrospective and anonymous nature of this study no link could be made with development of CIN at this stage. Future investigations are being initiated on the linkage of the anonymised hrHPV data with follow up data from the pathology registry database to enable the investigation of clinical relevance of concordant and discrepant hrHPV positive samples in the DuSC study.

## Conclusions

In conclusion, our data strongly suggest a higher hrHPV prevalence of approximately 8% in the Dutch cervical cancer screening population than the hrHPV prevalence of 4–5% that was generally used up to now. This higher prevalence was determined independently of the hrHPV assay used. The complete automated solutions evaluated will greatly facilitate the implementation of primary hrHPV testing in cervival cancer screening.

## Abbreviations

AIS: Adenocarcinoma in situ
ASC-US: Atypical squamous cells of unknown significance
HC2: Hybrid Capture 2
CIN: Cervical intraepithelial neoplasia
CIS: Carcinoma in situ
Cp value: Crossing point value
DuSC hrHPV study: Dutch screening comparison high risk human papillomavirus study
HrHPV: High risk human papillomavirus
HSIL: High-grade Squamous Intraepithelial Lesion
LSIL: Low-grade Squamous Intraepithelial Lesion
NVVP: Dutch society of pathology
RLU/CO: Relative light unit coefficient
UNG: Uracil N-glycosylase

## References

[1] Netherlands HCot. Population screening for cervical cancer. The Hague: Health Council of the Netherlands. 2011,publication no. 2011/07.

[2] Bulkmans NW, Berkhof J, Rozendaal L, van Kemenade FJ, Boeke AJ, Bulk S (2007). Human papillomavirus DNA testing for the detection of cervical intraepithelial neoplasia grade 3 and cancer: 5-year follow-up of a randomised controlled implementation trial. Lancet.

[3] Ronco G, Giorgi-Rossi P, Carozzi F, Confortini M, Dalla Palma P, Del Mistro A (2010). Efficacy of human papillomavirus testing for the detection of invasive cervical cancers and cervical intraepithelial neoplasia: a randomised controlled trial. Lancet Oncol.

[4] Ronco G, Dillner J, Elfstrom KM, Tunesi S, Snijders PJ, Arbyn M (2014). Efficacy of HPV-based screening for prevention of invasive cervical cancer: follow-up of four European randomised controlled trials. Lancet.

[5] Meijer CJ, Berkhof J, Castle PE, Hesselink AT, Franco EL, Ronco G (2009). Guidelines for human papillomavirus DNA test requirements for primary cervical cancer screening in women 30 years and older. Int J Cancer.

[6] Meijer CJ, Berkhof H, Heideman DA, Hesselink AT, Snijders PJ (2009). Validation of high-risk HPV tests for primary cervical screening. J Clin Virol.

[7] Rijkaart DC, Berkhof J, van Kemenade FJ, Coupe VM, Hesselink AT, Rozendaal L (2012). Evaluation of 14 triage strategies for HPV DNA-positive women in population-based cervical screening. Int J Cancer.

[8] Bulkmans NW, Rozendaal L, Snijders PJ, Voorhorst FJ, Boeke AJ, Zandwijken GR (2004). POBASCAM, a population-based randomized controlled trial for implementation of high-risk HPV testing in cervical screening: design, methods and baseline data of 44,102 women. Int J Cancer.

[9] Cook DA, Mei W, Smith LW, van Niekerk DJ, Ceballos K, Franco EL (2015). Comparison of the Roche cobas(R) 4800 and Digene Hybrid Capture(R) 2 HPV tests for primary cervical cancer screening in the HPV FOCAL trial. BMC Cancer.

[10] Preisler S, Rebolj M, Untermann A, Ejegod DM, Lynge E, Rygaard C (2013). Prevalence of human papillomavirus in 5,072 consecutive cervical SurePath samples evaluated with the Roche cobas HPV real-time PCR assay. PLoS One.

[11] Rijkaart DC, Berkhof J, van Kemenade FJ, Rozendaal L, Verheijen RH, Bulk S (2010). Comparison of HPV and cytology triage algorithms for women with borderline or mild dyskaryosis in population-based cervical screening (VUSA-screen study). Int J Cancer.

[12] Wright TC, Stoler MH, Behrens CM, Sharma A, Zhang G, Wright TL (2015). Primary cervical cancer screening with human papillomavirus: end of study results from the ATHENA study using HPV as the first-line screening test. Gynecol Oncol.

[13] Arbyn M, Snijders PJ, Meijer CJ, Berkhof J, Cuschieri K, Kocjan BJ (2015). Which high-risk HPV assays fulfil criteria for use in primary cervical cancer screening?. Clin Microbiol Infect.

[14] Erasmus MC MG (2011). Landelijke Evaluatie Bevolkingsonderzoek Baarmoederhalskanker.

[15] NVVP (2010). WMDP-richtlijn “Het gebruik van moleculaire HPV testen in het BVO” in het kader van de HPV diagnostiek bij preventie van baarmoederhalskanker.

[16] Landis JR, Koch GG (1977). The measurement of observer agreement for categorical data. Biometrics.

[17] Ogilvie GS, van Niekerk DJ, Krajden M, Martin RE, Ehlen TG, Ceballos K (2010). A randomized controlled trial of Human Papillomavirus (HPV) testing for cervical cancer screening: trial design and preliminary results (HPV FOCAL Trial). BMC Cancer.

[18] Rebolj M, Bonde J, Preisler S, Ejegod D, Rygaard C, Lynge E (2016). Human Papillomavirus Assays and Cytology in Primary Cervical Screening of Women Aged 30 Years and Above. PLoS One.

[19] Cuzick J, Cadman L, Mesher D, Austin J, Ashdown-Barr L, Ho L (2013). Comparing the performance of six human papillomavirus tests in a screening population. Br J Cancer.

[20] Getman D, Aiyer A, Dockter J, Giachetti C, Zhang F, Ginocchio CC (2009). Efficiency of the APTIMA HPV Assay for detection of HPV RNA and DNA targets. J Clin Virol.

[21] Heideman DA, Hesselink AT, Berkhof J, van Kemenade F, Melchers WJ, Daalmeijer NF (2011). Clinical validation of the cobas 4800 HPV test for cervical screening purposes. J Clin Microbiol.

[22] Lloveras B, Gomez S, Alameda F, Bellosillo B, Mojal S, Muset M (2013). HPV testing by cobas HPV test in a population from Catalonia. PLoS One.

[23] Wu R, Belinson SE, Du H, Na W, Qu X, Liu Y (2010). Human papillomavirus messenger RNA assay for cervical cancer screening: the Shenzhen Cervical Cancer Screening Trial I. Int J Gynecol Cancer.

[24] Rebolj M, Preisler S, Ejegod DM, Rygaard C, Lynge E, Bonde J (2014). Disagreement between human papillomavirus assays: an unexpected challenge for the choice of an assay in primary cervical screening. PLoS One.

[25] Levi AW, Bernstein JI, Hui P, Duch K, Schofield K, Chhieng DC (2016). A Comparison of the Roche Cobas HPV Test With the Hybrid Capture 2 Test for the Detection of High-Risk Human Papillomavirus Genotypes. Arch Pathol Lab Med.

[26] Flores R, Papenfuss M, Klimecki WT, Giuliano AR (2006). Cross-sectional analysis of oncogenic HPV viral load and cervical intraepithelial neoplasia. Int J Cancer.

[27] Haedicke J, Iftner T (2016). A review of the clinical performance of the Aptima HPV assay. J Clin Virol.

[28] Fornari D, Rebolj M, Bjerregard B, Lidang M, Christensen I, Hogdall E (2016). Hybrid Capture 2 and cobas human papillomavirus assays perform similarly on SurePath samples from women with abnormalities. Cytopathology.

